# Impact of job resources on organizational citizenship behavior among primary care staff in Guangzhou, China: a cross-sectional study

**DOI:** 10.3389/fpubh.2025.1580148

**Published:** 2025-06-20

**Authors:** Liying Fan, Jiamin Yang, Qiao Hou, Shanshan Feng, Aiyun Chen, Lingzhi Han

**Affiliations:** ^1^School of Health Management, Guangzhou Medical University, Guangzhou, Guangdong, China; ^2^Social Science Key Laboratory of Guangdong Higher Education Institutes for Health Governance Based on Big Data Utilization, Guangzhou, Guangdong, China

**Keywords:** primary care staff, job resources, organizational citizenship behavior, demands abilities fit, work motivation

## Abstract

**Background:**

Low motivation to work among primary care staff is the key hindrance to the development of primary healthcare. Job resources are a kind of tool that helps employees achieve their work objectives and an internal motivating factor that generates positive behavior. This study investigates the impact of job resources on organizational citizenship behavior (OCB) among primary care staff and the moderating effect of demands-abilities fit on this relationship. The ultimate goal is to improve primary care staff’s OCB levels and stimulate their motivation at work to provide policy suggestions.

**Methods:**

Taking Guangzhou City in southern China as the research site, this cross-sectional study selected 600 primary care staff using the multi-stage stratified random sampling method. The job resource scale, OCB scale, and demands-abilities fit scale were used as research instruments. Data were collected from September to November 2019 using a self-filling method. The multiple linear regression method was used to analyze the influence of job resources on OCB. The hierarchical multiple regression analysis was used to examine the moderating effect of demands-abilities fit.

**Results:**

Overall, 512 effective questionnaires were completed, with a recovery rate of 85.3%. Multiple linear regression analysis, after controlling for socio-demographic factors, shows job resources (*β* = 0.489, *p* < 0.001) and three dimensions of job resources: Social support (*β* = 0.175, *p* < 0.01), job control (*β* = 0.177, *p* < 0.01), and possibilities for professional development (*β* = 0.203, *p* < 0.001) had a positive predictive effect on OCB. Demands-abilities fit moderated the positive predictive effects of job resources (*β* = 0.095, *p* < 0.01), social support (*β* = 0.077, *p* < 0.05), job control (*β* = 0.102, *p* < 0.01), and possibilities for professional development (*β* = 0.113, *p* < 0.01) on OCB.

**Conclusion:**

The findings demonstrated that the higher the level of job resources perceived by primary care staff, the higher the OCB; furthermore, the demands-abilities fit enhances the positive effect of job resources on OCB. It is suggested that primary healthcare institutions should prioritize creating an organizational atmosphere of interpersonal support, appropriately ensure the autonomy of primary care staff in their work, and pay attention to the needs of professional development and ability improvement. These measures may improve the OCB of primary care staff, stimulate their internal motivation, and provide residents with good primary health services.

## 1 Background

Primary healthcare is a crucial part of the health system, and a good primary healthcare system often has better healthcare quality and equity (1). Primary care staff are the healthcare system’s most critical and active core resources. However, low enthusiasm among primary care staff has become a key factor hindering the development of primary healthcare institutions, especially in low and middle-income countries (2). Therefore, the question of how to stimulate primary care staff’s enthusiasm has become the focus of governments and scholars.

The performance of primary care staff is difficult to measure effectively using explicit performance indicators, as it has internal recessive and non-economic characteristics. The enthusiasm of primary care staff should not only be motivated by pay, but also by stimulating their subjective initiative. Scholars refer to this kind of employee ownership behavior as organizational citizenship behavior (OCB) (3). This positive work behavior is not included in formal reward, punishment, or salary systems; however, it can improve the performance of individuals or organizations, manifesting in helping others, doing due diligence (4), taking the initiative to safeguard the organization’s interests, and other behaviors. If primary care staff have good OCB, they will be more willing to spend time and energy on organizational development to improve the organizations’ performance, optimize patient-centered behaviors, and enhance service quality (5). Additionally, they will help reduce the incidence of adverse events (6), ultimately fostering the health of the residents even more. OCB of primary care staff is crucial in enhancing their motivation to work and in improving the performance of primary health services.

In research on the factors influencing OCB, job characteristics are considered important predictors. Van den Broeck et al. (7) divided job characteristics into job resources, hindrances, and challenges, among which job resources positively affect employees’ behaviors. Bakker (8) believes that job resources are significant predictors of out-of-role behaviors. Job resources help employees achieve their work goals and are therefore an internal motivating factor that generates positive motivation and behavior and an important factor promoting the formation of OCB (9). According to social exchange theory (SET), employees reciprocate toward the resource provider, and are affected by work ability, work progress, and other factors; employees are more willing to choose positive behaviors not stipulated by the reward and punishment system, that is, to practice OCB as a set of “rewards” (10), such as being willing to help colleagues, taking the initiative to undertake work responsibilities, and providing active input. Although evidence suggests associations between leadership/team-member exchange, job autonomy, and career development in job resources with the improvement of OCB (11–13), research that combines these three variables remains limited. Academic exploration into the connection between multidimensional job resources and OCB in healthcare settings is insufficient.

China’s new healthcare reform in 2009 identified a health reform strategy “focusing on primary care,” which carried out a series of primary health reform measures, such as equalizing basic public health services, family doctor contract services, and medical treatment combinations (14, 15). Primary care staff’s work tasks have been continuously diversified, and their work roles have been continuously reshaped and expanded. Higher work requirements are put forward for primary care staff. The main dilemma faced by current primary care staff is high job demands, which further causes the lack of motivation to work and reduces the vitality of primary care staff; this is an important reason hindering the development of primary medical and health institutions (16). High job demands pose new challenges to the abilities of primary care staff, and demands-abilities fit refers to the degree of alignment between individual abilities and job demands. Against this backdrop, the impact of the degree to which primary care staff’s demands-abilities fit affects their behavior has become an urgent issue to be explored.

Therefore, this study intends to explore the impact of job resources on the OCB perceived by primary care staff in Guangzhou, southern China, as well as the moderating role of demands-abilities fit. Furthermore, it aims to provide a theoretical and practical foundation for stimulating the initiative of primary care staff and improving their motivation to work. As a developing country, research results from China are a valuable reference for most developing countries seeking to enhance the motivation of primary care staff.

## 2 Literature review and research hypotheses

Organ (4) proposed the concept of OCB and presented a specific definition: “a kind of behavior that is carried out spontaneously by employees, outside the reward system but is beneficial to organizational performance on the whole” (4). Organ proposed a five-dimensional classification model for OCB, including civic morality, altruism, sportsmanship, sense of responsibility, and courtesy. The measurement scale based on the five-dimensional structure became the basis for many scholars to compile later OCB scales (17). After researching the three dimensions of altruism, conscientiousness, and voice, Farh et al. (10) proposed a measurement scale, applicable to the Chinese context based on these three dimensions, which possesses strong reliability and validity and has been extensively utilized (18). A three-dimensional model was used in this study, in which altruism refers to willingness to help colleagues solve work problems or other personal problems; conscientiousness refers to commitment to work and the initiative to take responsibility, such as working overtime and cooperating with colleagues. Voice refers to employees’ proactive behavior in identifying problems and offering constructive suggestions for organizational improvement. Job resources encompass the physical, social, and organizational work factors that promote the completion of work objectives, reduce work requirements and psychological and physical losses, and facilitate personal growth and development. Some examples are job control, feedback, development opportunities (learning and promotion), social support, salary, and welfare. Job resources stimulate motivation and bring high job involvement and excellent job performance (8). The widely recognized research by Bakker and Demerouti et al. divides job resources into three dimensions: social support (interpersonal level), job control (task level), and possibilities for professional development (organizational level). Social support is subjective or objective support from family, leaders, and organizations. Job control represents the autonomy and decision-making power of employees at work. Possibilities for professional development refer to the opportunities for career development and self-ability improvement provided by the organization, as perceived by employees (19).

Organ believes that SET is an important theoretical basis for the OCB concept and that when employees perceive managers’ efforts as achieving beneficial results, they will be inclined toward giving back to managers (4). Homans believes that in addition to the traditional economic exchange relationship, there is a non-material exchange relationship between people, which is referred to as social exchange (20). The principle of reciprocity is core, wherein individuals feel obligated to reciprocate when they perceive respect and effort from others. When primary care staff receive ample job resources, they are inclined to reciprocate by showing appreciation toward their managers or organizations that provide these resources. Owing to limitations in their abilities and work processes, organizational members may find it difficult to reciprocate by increasing their work output or improving efficiency. Instead, they demonstrate OCB as a way of reciprocating toward the organization. Therefore, this paper proposes the following hypothesis:

*H1:* Job resources have a positive predictive effect on organizational citizenship behavior.

According to SET and the principle of reciprocity, when a favor is given, recipients have positive emotions and want to return the favor (21). Receiving support from the organization, leaders, teams, and colleagues is conducive to forming high-quality social-exchange relationships. Employees will put forth behaviors that benefit the organization, leaders, teams, and colleagues, promoting the generation of extra-role behaviors among organization members and forming OCB. Empirical studies have shown that organizational members’ perceived support from leaders can promote members’ OCB; in return, members make efforts beyond the scope of their responsibilities, showing their conscientiousness and altruism (22, 23). Empirical studies have confirmed that social support is an important predictor of altruism (24). Simultaneously, some studies have shown that support from organizations, leaders, and colleagues, is significantly positively correlated with voice, causing organization members to practice more promoting and inhibiting forms of voice (25, 26).

On the one hand, the perceived support and high-quality social exchange relationship formed by organizational members can increase their perception of the positive effect of voice. On the other hand, it can reduce their perception of the potential risks of voice behavior and increase their awareness of its positive results, thus promoting voice behavior (27). Therefore, this paper proposes the following hypothesis.

*H1a:* Social support has a positive predictive effect on OCB.

Primary care staff are considered knowledge workers, and tapping into their initiative and enthusiasm hinges on internal motivation. Compared with other types of employees, knowledge workers especially emphasize autonomy, competence, and relatedness between three kinds of inner psychological needs. Furthermore, the level of job control is key to meeting their job autonomy and competence needs, reflecting managers’ authorization behavior in practice. When members of the organization have enough freedom to decide and master their work methods and processes, knowledge workers often gain more interest in work, a sense of participation, job security, and a sense of responsibility for their work. These positive emotions promote the generation of employee OCB (28–30). Employees with high job autonomy are more likely to exhibit OCB than those with low job autonomy (31). Shujie (32) also found that job autonomy positively promotes organizational citizenship behavior in a study on university administrators. After constructing the research framework of OCB, Van Dyne et al. (33) proposed that high job autonomy gives employees a stronger sense of responsibility for their work results and organizational development, thus promoting the generation of their OCB. When knowledge workers perceive autonomy support in their work, they are more willing to voice their opinions within the organization (34). Therefore, this study proposes the following hypothesis:

*H1b:* Job control has a positive predictive effect on OCB.

Primary care staff often have strong growth expectations. When organizations provide career development and training opportunities for employees, their growth expectations are largely met and become the internal motivating factors for their OCB (35). Meanwhile, good development opportunities may signal that the organization pays attention to employees. Following the principle of reciprocity, employees view training opportunities, chances for promotion, and favorable career development prospects as indications that the organization values them enough to invest in their growth. This positive treatment fosters high-quality social exchange relationships with the organization, ultimately stimulating OCB among employees. Whittington believes that appropriate human resource management practices can promote organization members to show OCB. When they perceive that the organization provides them with good career and ability development opportunities, the attitude of the organization members toward their work will be significantly improved. Thereafter, the level of OCB will be enhanced, reflecting the reciprocal behavior norms (36, 37). Venkataramani and Tangirala (38) have proven that employees with good career development have more voice initiative. Therefore, this study proposes the following hypothesis:

*H1c:* Possibilities for professional development have a positive predictive effect on OCB.

Demands-abilities fit refers to the degree to which job demands fit the skills and abilities of employees (39). A high consistency between job demands and a person’s abilities will lead to better job performance (40). When employees’ job demands match their abilities, they have better competence, the working environment is conducive to their skills and abilities, and they will be willing to put in more time and energy at work (41). Self-determination theory (SDT) holds that individuals have basic autonomy, relatedness, and competence requirements. These three psychological needs are universal and innate. Individuals tend to be close to the environment if the environment can fulfill these needs and try their best to meet these three needs. When these three innate needs are met, individuals will be driven by their intrinsic work motivation to exert their potential and produce positive work results (42). SDT elucidates how an individual’s intrinsic motivation can create psychological incentives for employees upon fulfilling their needs. According to the Job Demands-Resources model, abundant job resources provide a working environment conducive to developing employees’ skills and abilities. When individuals perceive a higher level of matching between themselves and the demands of the job they undertake, they may generate more positive emotions, further strengthen the gain path of job resources, and, in the process, become dependent and belong to the organization. When the primary care staff perceives that their ability fits the job demands, they meet their psychological needs for competence, develop work incentives, and better play the role of job resources, strengthening their sense of belonging to the organization and enhance their OCB. Relevant studies on the demands-abilities fit of primary care staff at home and abroad are lacking. This study takes the demands-abilities to fit as a moderating variable and proposes the following research hypothesis:

*H2:* The demands-abilities fit moderates job resources and the OCB of primary care staff; that is, the demands-abilities fit enhances the positive effect of job resources on OCB.

### 2.1 Conceptual model

**Figure 1:**
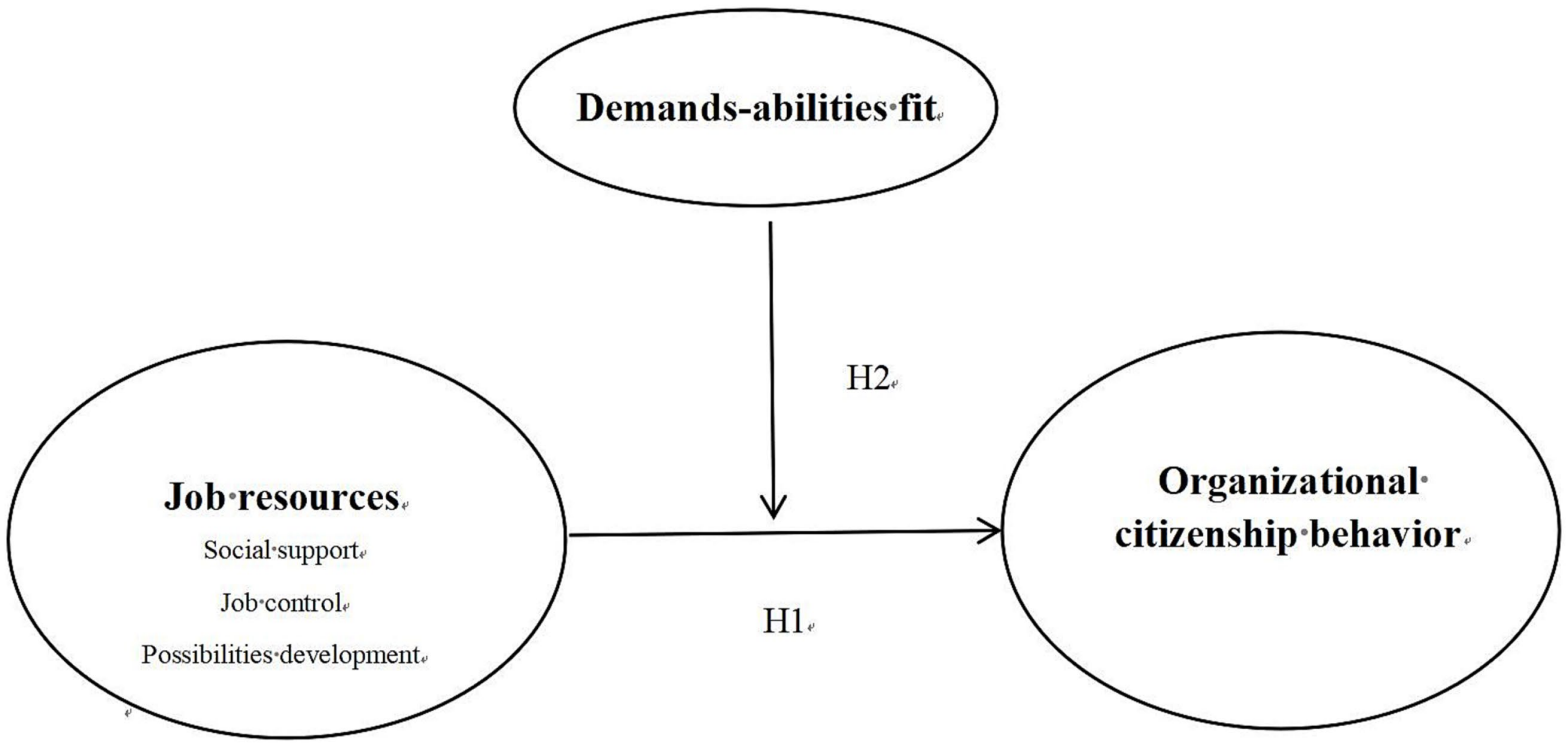
Conceptual model.

Based on the preceding literature review concerning SET and SDT, the conceptual model is presented in Figure 1. First, we examine the direct relationship between job resources and OCB. Second, we test the mediating effect of Demands-abilities fit on the relationship between job resources and OCB.

## 3 Methods

### 3.1 Study design and sample

This cross-sectional study was conducted in Guangzhou, South China’s Guangdong Province, from September to November 2019, using a multistage sampling method to select samples. In the initial stage, 11 districts in Guangzhou were categorized into three groups based on their economic status, and one district was randomly selected from each group—a total of three districts. In the second stage, four community health centers (CHCs) were randomly selected in each district, and 12 CHCs were used as investigation institutions. In the third stage, each CHC surveyed 50 primary care staff, including on-the-job clinicians, public health doctors, and nurses, up to a total of 600 participants. The following inclusion criteria were used: (1) the study participants must have signed the informed consent and understood the questionnaire content clearly; (2) the study participants must have signed labor contracts with primary healthcare institutions; and (3) the study participants must have worked in a primary medical institution for over half a year.

### 3.2 Data collection process

We enlisted graduate and undergraduate students from Guangzhou Medical University as investigators. To ensure the accuracy and consistency of the survey, we trained the investigators on investigation techniques, including how to explain the purpose of the survey and the meaning of each item in the questionnaire. Respondents signed informed consent forms prior to the survey visit, which clearly stated the principle of voluntary participation. With assistance from managers at the CHCs, self-administered questionnaires were distributed to primary care staff at these institutions. Respondents received souvenirs for participating in the survey. In total, 600 questionnaires were collected, and careful screening was conducted to eliminate any incomplete or disorderly filled-in questionnaires.

In this study, 62 primary care staff from different CHCs were randomly selected for pre-investigation. The sample standard deviation *σ* was 0.56, the allowable error *δ* was 0.071, and the test level *α* was set as 0.05. The sample size was calculated as *n* = [(*u*_*α*/2_*σ)/*δ*]^2^ = 239; considering the invalid questionnaire, the sample size was expanded by 20%, and the minimum sample size was approximately 287. This study’s valid samples were 512 and over 287, sufficient to provide good statistical efficacy.

### 3.3 Instruments

This study referred to the existing international maturity scale, translated by members of the research group after authorization by the author of the original scale, and modified the questionnaire after consultation with English experts and conducting a pre-survey. Data were collected using a survey questionnaire that consisted of four sections: sociodemographic data and three scales. Sociodemographic data includes gender, marital status, education, authorized strength, age, type of job, and professional title. The three scales include the OCB Scale, job resource scale, and demands-abilities fit scale.

#### 3.3.1 OCB scale

The People’s Republic of China OCB Scale (PRC OCB Scale) developed by Dr. Farh of the Hong Kong University of Science and Technology was adopted to measure the OCB status of primary care staff (10). The scale measures OCB through 3 dimensions: altruism, voice, and conscientiousness, with nine items. The items in the questionnaire are rated on a 5-point Likert scale ranging from 1 (completely inconsistent) to 5 (completely consistent), with higher scores indicating higher levels of OCB. Previous studies have demonstrated that this scale exhibits good reliability and validity.

The Cronbach’s alpha (*α*) coefficient and Kaiser-Meyer-Olkin (KMO) of the OCB scale measured in this study were 0.865 and 0.856, respectively.

#### 3.3.2 Job resource scale

We used the scale developed by Bakker et al. (19) and the scale of job resources developed by Lusheng (43) to divide job resources into three dimensions: social support, possibilities for professional development, and job control, with 12 items. The items were assessed using a 5-point Likert scale, with 1 indicating complete inconsistency and 5 indicating complete consistency. A higher score indicates greater job resources. The job resource scale measured in this study has good reliability and validity, with the Cronbach’s *α* coefficient being 0.905 and KMO being 0.831.

#### 3.3.3 Demands-abilities fit scale

We used Cable et al.’s (44) three-item scale, which comprises “Job demands are well matched with my skills,” “My ability and training are fully able to meet the demands of the job,” and “My ability and education are well matched with the demands of the job.” The items are rated on a 5-point Likert scale, with 1 being completely inconsistent and 5 being completely consistent. A higher score reflects a better demands-abilities fit degree.

### 3.4 Data analyses

Following the interviews, all questionnaires were double-checked for accuracy. Data documentation was carried out using Epidata 3.1 by two independent students, followed by crosschecking after data input. Analysis was performed using SPSS 22.0 software; descriptive statistics were initially utilized to analyze social demographics within our samples as well as basic information related to OCB. The study utilized Harman’s single-factor test to examine common method bias, the Pearson correlation test was used to examine the relationship between job resources, demands-abilities fit, and OCB. Additionally, multiple linear regression was employed to analyze the association between job resources and OCB, and hierarchical multiple regression was used to examine the moderating effect of demands-abilities fit. Based on the simple slope test, interaction plots were drawn to further predict and validate the moderating effect.

## 4 Results

A total of 600 primary care staff were included in the study, 512 of whom completed the questionnaire, with a response rate of 85.3%.

### 4.1 Demographic characteristics

Five hundred twelve primary care staff in Guangzhou ranged in age from 20 to 57, 77.15% of them were women, and 69.53% were married. Regarding education, the majority had a bachelor’s degree or above (75.97%). Regarding work, the primary care staff interviewed were mainly nurses (41.99%), the number with authorized strength was slightly higher than that of staff without authorized strength (51.76%), and the number of staff with junior titles was slightly higher than that of staff with middle and senior titles (53.71%).

The scores of OCB of primary care staff with different marital statuses, authorized strength, age, and professional titles were statistically significant (*p* < 0.05). Married people have authorized strength; the older they are, the higher their professional titles, and the higher their OCB. See Table 1.

**Table 1:** Comparison of OCB of primary care staff with different social demographic characteristics (*n* = 512).

Variable	Total (NO.%)	0CB (s)	*t*/*F*值
Sociodemographic characteristics			
Gender			−1.616
Male	117 (22.85)	3.84 (0.61)	
Female	395 (77.15)	3.93 (0.51)	
Marital status			−3.504***
Yes	156 (30.47)	3.78 (0.52)	
No	356 (69.53)	3.96 (0.53)	
Education			−0.448
Junior college and below	123 (24.03)	3.89 (0.56)	
Bachelor’s degree or above	389 (75.97)	3.91 (0.53)	
Authorized strength			−3.735***
Yes	247 (48.24)	3.82 (0.57)	
No	265 (51.76)	3.99 (0.48)	
Age			−4.554***
<34	270 (44.12)	3.81 (0.51)	
≥34	242 (39.54)	4.02 (0.55)	
Type of job			1.337
Clinical and TCM doctors	199 (38.87)	3.87 (0.55)	
Public health doctor	98 (19.14)	3.88 (0.5)	
Nurse	215 (41.99)	3.95 (0.54)	
Professional title			−3.996***
Primary title	275 (53.71)	3.82 (0.56)	
Senior title	237 (46.29)	4.01 (0.49)	

***p < 0.001.

### 4.2 Common method bias test

Common method bias was tested using Harman’s single-factor analysis. All variables to be analyzed were included in the exploratory factor analysis without rotation, and the results of the unrotated principal component analysis were examined. The test results showed that there were five factors with eigenvalues greater than 1, and the variance contribution rate of the first factor was 39.29%, which did not exceed the criterion of 50% (45). Therefore, common method bias is not a concern in this study.

### 4.3 Correlation analysis of job resources, demands-abilities fit, and OCB

The average scores of perceived job resources among primary care staff in Guangzhou were 3.61, with mean scores for social support, job control, and possibilities for professional development being 3.85, 3.43, and 3.53, respectively. and the lowest job control score was. The overall score of demands-abilities fit was 3.89, and the overall score of OCB was 3.91. The results showed that job resources (*r* = 0.468, *p* < 0.01) and its three dimensions—social support (*r* = 0.379, *p* < 0.01), job control (*r* = 0.413, *p* < 0.01), and possibilities for professional development (*r* = 0.450, *p* < 0.01) were positively correlated with OCB. Demands-abilities fit was positively correlated with OCB (*r* = 0.532, *p* < 0.01) (Table 2).

**Table 2:** Correlation of job resources, demands-abilities fit, and OCB of primary care staff (*n* = 512).

Variables	Score ( $\bar{x}$ ± s)	Correlation coefficient (*r*-value)					
		OCB	Job resources	Social support	Job control	Possibilities development	Demands-abilities fit
OCB	3.91 ± 0.53	1		–	–	–	–
Job resources	3.61 ± 0.68	0.468**	1				
Social support	3.85 ± 0.69	0.379**	0.866**	1	–	–	–
Job control	3.43 ± 0.77	0.413**	0.900**	0.652**	1	–	–
Possibilities development	3.53 ± 0.87	0.450**	0.880**	0.646**	0.705**	1	–
Demands-abilities fit	3.89 ± 0.70	0.532**	0.354**	0.302**	0.334**	0.297**	1

***p < 0.001.

### 4.4 Impact of job resources on OCB

Multiple linear regression analysis was conducted with OCB as the dependent variable, job resources as the independent variable, and socio-demographic variables as control variables. The results showed that job resources (*β* = 0.489, *p* < 0.001) significantly and positively predicted OCB. Furthermore, the results showed that after controlling for socio-demographic variables, job resources significantly and positively predicted altruistic behavior (*β* = 0.440, *p* < 0.001), voice (*β* = 0.414, *p* < 0.001), and conscientiousness (*β* = 0.394, *p* < 0.001). The change in R-squared (Δ*R*^2^ = 0.291–0.054 = 0.237) indicates that job resources can explain 23.7% of the variance in OCB, as presented in Tables 3, 4.

**Table 3:** Results of multiple linear regression analysis of job resources on organizational citizenship behavior and its dimensions.

Variables	OCB			Dimensions of OCB								
				Altruism			Voice			Conscientiousness		
	*B*	*β*	*t*	*B*	*β*	*t*	*B*	*β*	*t*	*B*	*β*	*t*
Constant	1.977		14.390***	2.349		15.100***	1.917		10.733***	1.728		9.580***
Independent variables												
Job resources	0.385	0.489	13.020***	0.370	0.440	11.063***	0.398	0.414	10.358***	0.389	0.394	10.029***
Control variables												
Authorized strength	0.109	0.102	2.433*	0.075	0.065	1.437	0.132	0.101	2.277*	0.123	0.091	2.090*
Age	0.151	0.142	3.109**	0.035	0.031	0.635	0.095	0.073	1.504	0.267	0.199	4.174***
Professional title	0.100	0.093	1.990*	0.107	0.093	1.882	0.102	0.078	1.564	0.094	0.070	1.419
*R* ^2^	0.291			0.206			0.198			0.225		
*F*	51.990			32.791			31.255			36.766		
*p*	<0.001			<0.001			<0.001			<0.001		

***p < 0.001.

**p < 0.01.

*p < 0.05.

**Table 4:** Demands-abilities fit moderating effect results between job resources and OCB.

Variables	OCB (*β*)								
	Model 1	Model 2a	Model 2b	Model 2c	Model 2d	Model 3a	Model 3b	Model 3c	Model 3d
Control variables									
Authorized strength	0.089	0.111**	0.129**	0.113**	0.084*	0.115**	0.132**	0.118**	0.087*
Age	0.140**	0.074	0.091*	0.063	0.050	0.063	0.081	0.051	0.042
Professional title	0.059	0.093*	0.078	0.094*	0.091*	0.099*	0.082	0.101*	0.098*
Independent variables									
Job resources		0.348***				0.332***			
Social support			0.288***				0.287***		
Job control				0.292***				0.264***	
Possibilities development					0.319***				0.306***
Moderator variable									
Demands-abilities fit		0.391***	0.425***	0.418***	0.423***	0.420***	0.437***	0.455***	0.458***
Interaction terms									
Job resources × Demands-abilities fit						0.095**			
Social support × Demands-abilities fit							0.077*		
Job control × Demands-abilities fit								0.109**	
Possibilities development × Demands-abilities fit									0.113**
*R* ^2^	0.054	0.420	0.389	0.391	0.408	0.428	0.394	0.401	0.420
Δ*R*^2^	0.054	0.367	0.335	0.337	0.355	0.008	0.006	0.010	0.012
*F*	9.619***	73.393***	64.346***	64.889***	69.851***	63.105***	54.805***	56.364***	60.949***

***p < 0.001.

**p < 0.01.

*p < 0.05.

### 4.5 Impact of three dimensions of job resources on OCB

Multiple linear regression analysis was conducted with OCB as the dependent variable. Three dimensions of job resources—social support, job control, and possibilities for professional development—were used as the independent variables, and socio-demographic variables as the control variables. The results showed that the three dimensions of job resources—social support (*β* = 0.175, *p* < 0.01), job control (*β* = 0.177, *p* < 0.01), and possibilities for professional development (*β* = 0.203, *p* < 0.001) significantly positively predicted OCB. Further analysis was conducted on the impact of job resources on the three dimensions of OCB. The results showed that after controlling for socio-demographic variables, social support (*β* = 0.327, *p* < 0.001). Positively predicted altruistic behavior. Social support (*β* = 0.152, *p* < 0.01) and job control (*β* = 0.252, *p* < 0.001) both positively predicted voice. Job control (*β* = 0.132, *p* < 0.05) and possibilities for professional development (*β* = 0.281, *p* < 0.001) positively predicted conscientiousness, as shown in Table 5.

**Table 5:** Results of multiple linear regression analysis of various dimensions of job resources on organizational citizenship behavior and its dimensions.

Variables	OCB			Dimensions of OCB								
				Altruism			Voice			Conscientiousness		
	*B*	*β*	*t*	*B*	*β*	*t*	*B*	*β*	*t*	*B*	*β*	*t*
Constant	1.996		13.311***	2.165		12.855***	1.877		9.643***	1.930		9.871***
Independent variables												
Social support	0.135	0.175	3.232**	0.270	0.327	5.775***	0.143	0.152	2.653**	0.029	0.030	0.528
Job control	0.123	0.177	3.108**	0.072	0.097	1.631	0.214	0.252	4.175***	0.115	0.132	2.233*
Possibilities development	0.125	0.203	3.514***	0.052	0.079	1.311	0.048	0.063	1.034	0.217	0.281	4.703***
Control variables												
Authorized strength	0.105	0.098	2.316*	0.093	0.082	1.831	0.142	0.109	2.409*	0.096	0.071	1.616
Age	0.149	0.140	3.032**	0.057	0.050	1.028	0.099	0.076	1.549	0.244	0.182	3.798***
Professional title	0.100	0.093	1.980*	0.098	0.086	1.740	0.104	0.079	1.593	0.099	0.073	1.501
*R* ^2^	0.291			0.219			0.202			0.236		
*F*	34.604			23.663			21.255			26.004		
*p*	<0.001			<0.001			<0.001			<0.001		

***p < 0.001.

**p < 0.01.

*p < 0.05.

### 4.6 The moderating effect of demands-abilities fit between job resources and OCB

Hierarchical regression analysis was employed to examine the moderating effect of demands-abilities fit between job resources and OCB. First, all variables were mean-centered. Using OCB as the dependent variable, hierarchical regression analysis was conducted: Model 1 introduced control variables; Model 2 separately incorporated the main effects of job resources and demands-abilities fit (Model 2a), social support and demands-abilities fit (Model 2b), job control and demands-abilities fit (Model 2c), and possibilities for professional development and demands-abilities fit (Model 2d); Model 3 separately introduced the interaction terms “job resources × demands-abilities fit” (Model 3a), “social support × demands-abilities fit” (Model 3b), “job control × demands-abilities fit” (Model 3c), and “possibilities for professional development × demands-abilities fit” (Model 3d), as shown in Table 4.

**Figure 2:**
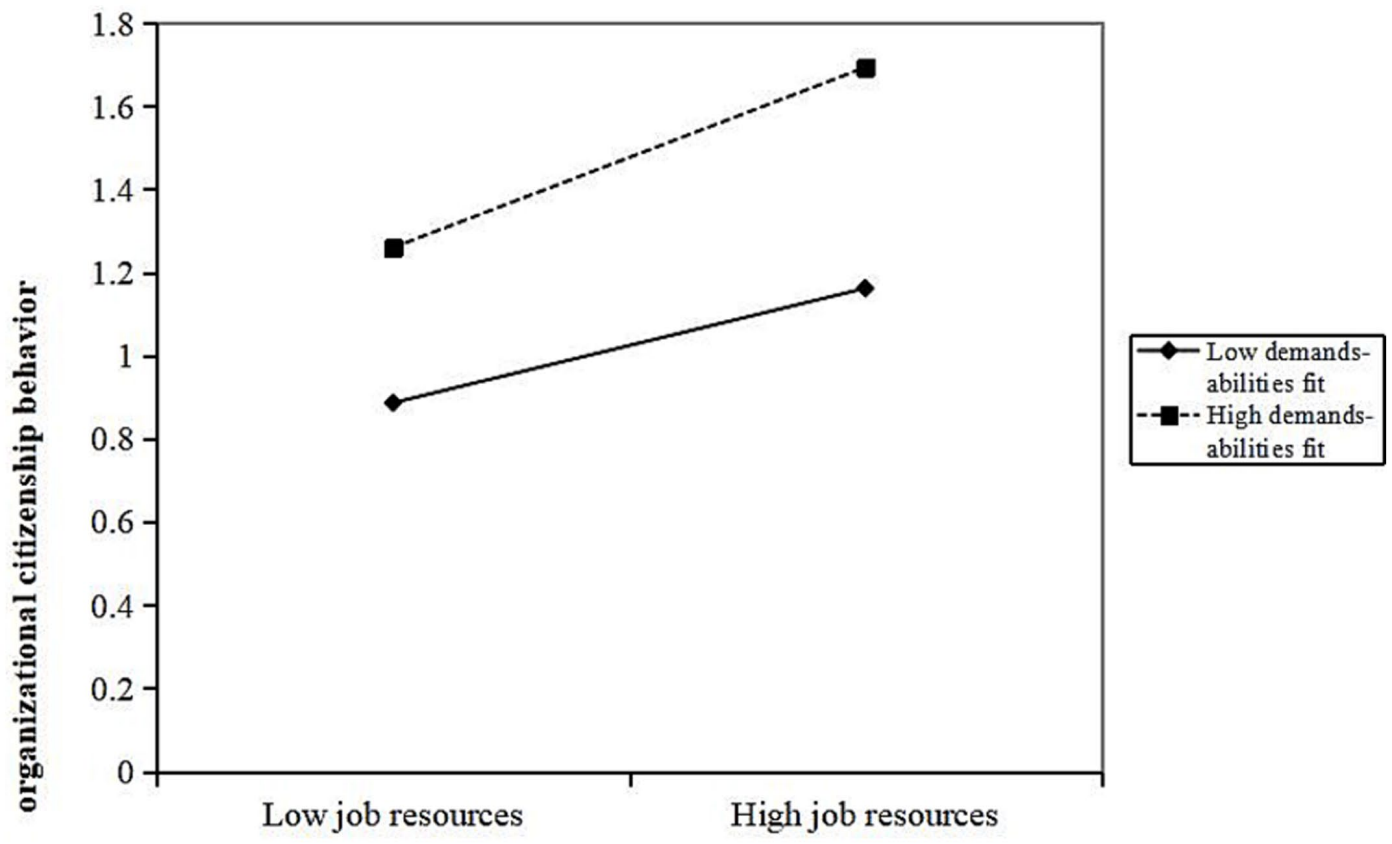
Moderating effect of demands abilities fit in the relationship between job resources and organizational citizenship behavior.

The results showed that demands-abilities fit has a moderating effect between job resources and OCB (*β* = 0.095, *p* < 0.01). After introducing the interaction term “job resources × demands-abilities fit,” the variance explained in OCB increased by 0.8% (Δ*R*^2^ = 0.008). When demands-abilities fit is high, it strengthens the positive promoting effect of job resources on OCB. As shown in Figure 2, compared to primary care staff with low demands-abilities fit, those with high demands-abilities fit exhibit a faster increase in OCB as job resources grow.

**Figure 3:**
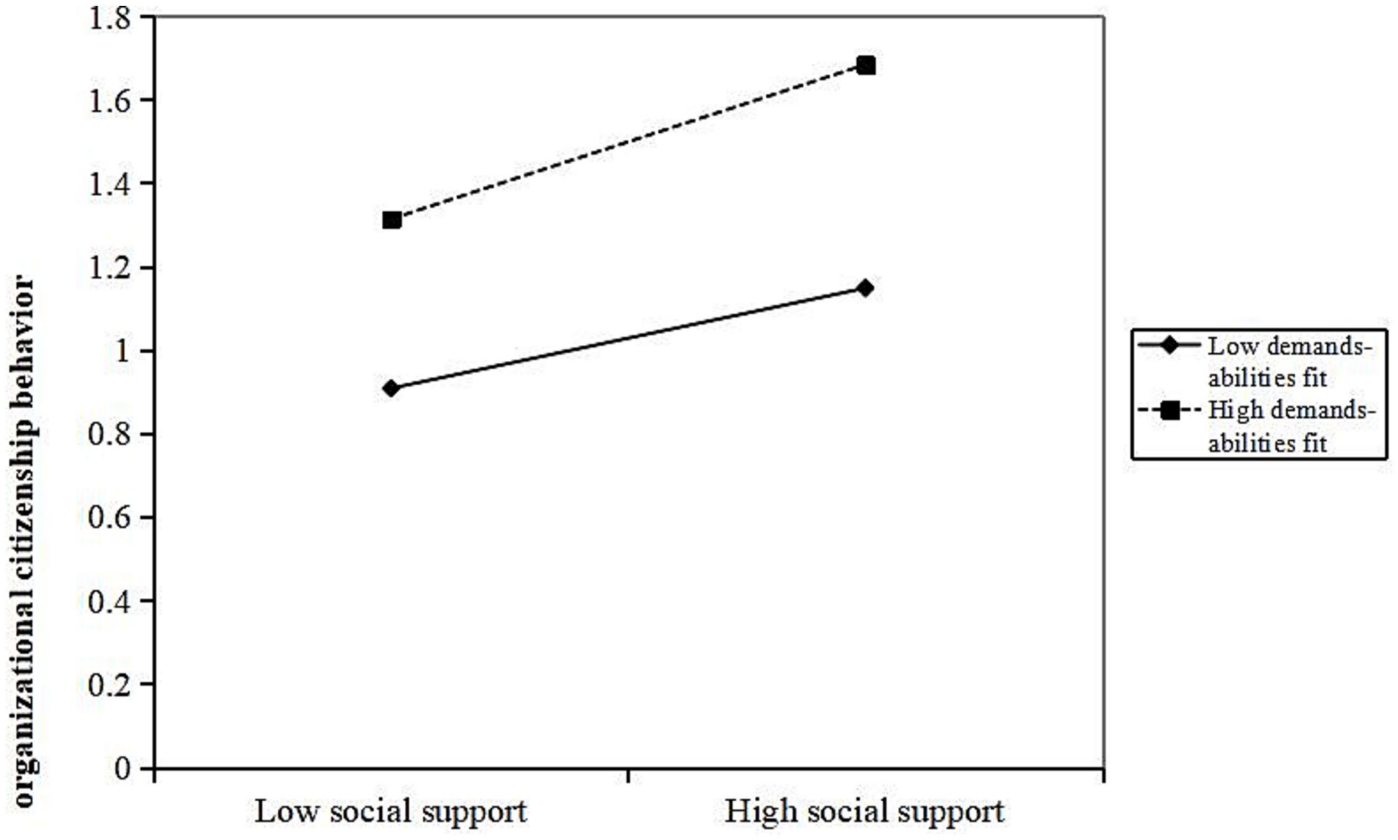
Moderating effect of demands abilities fit in the relationship between social support and organizational citizenship behavior.

**Figure 4:**
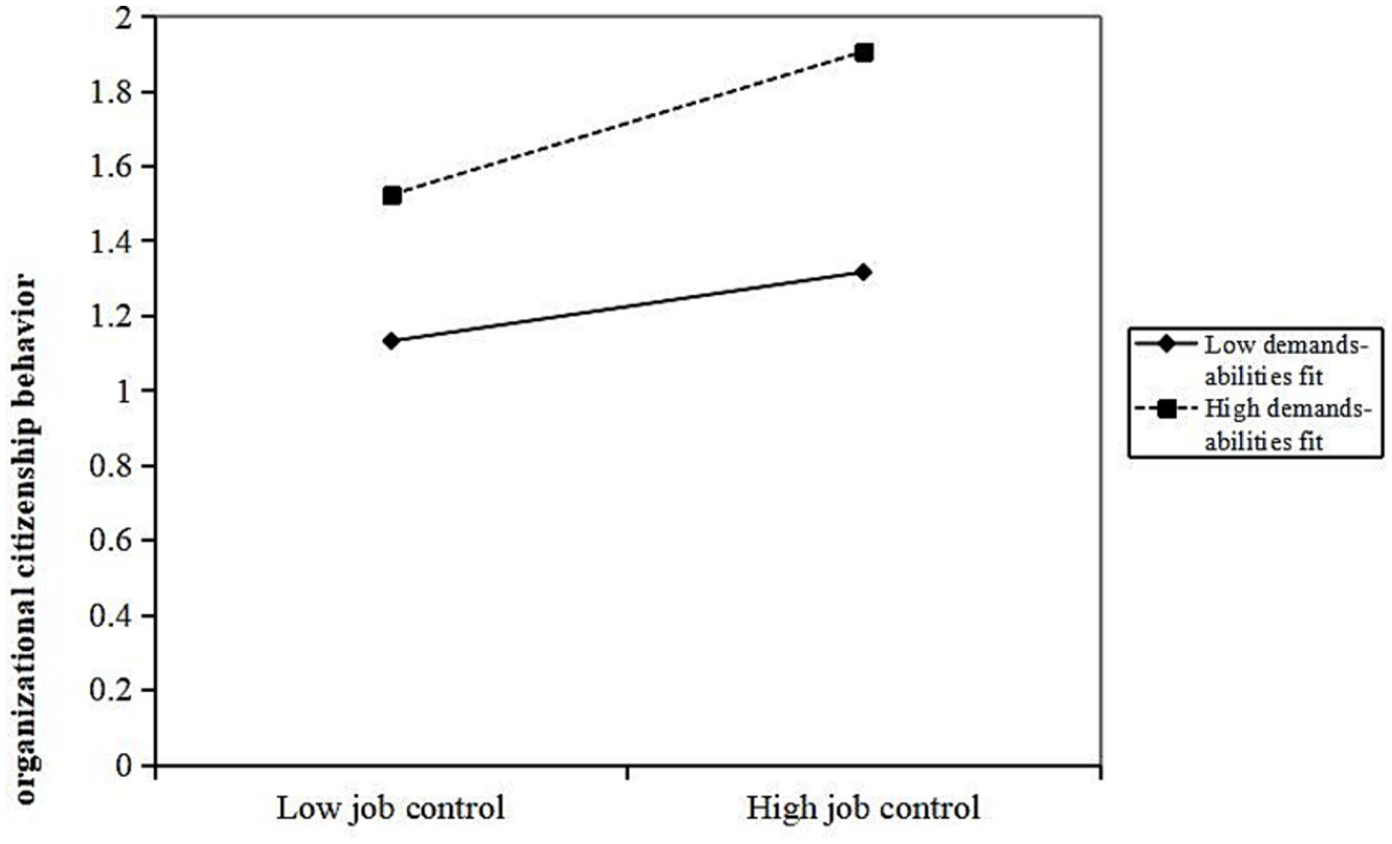
Moderating effect of demands abilities fit in the relationship between job control and organizational citizenship behavior.

**Figure 5:**
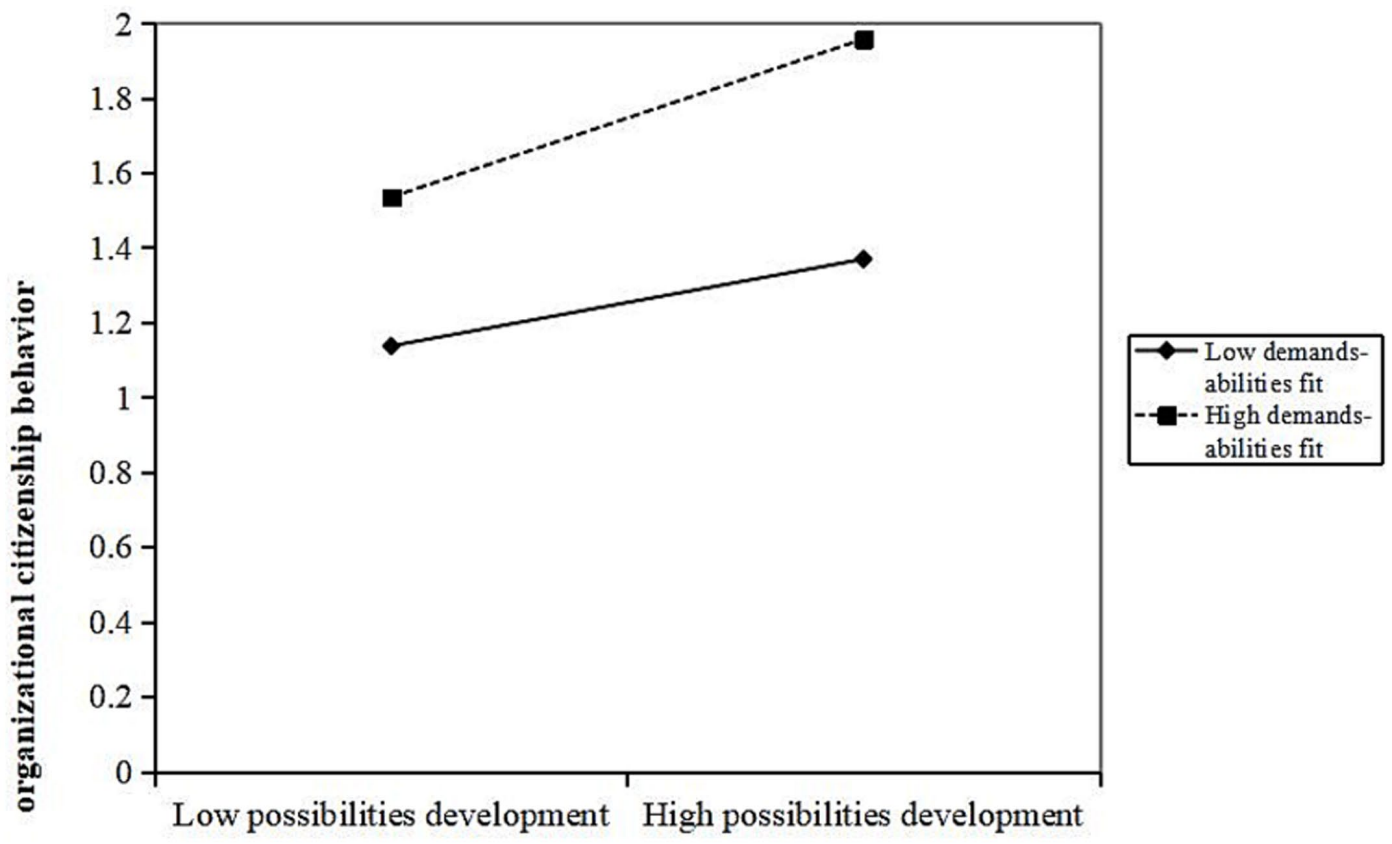
Moderating effect of demands abilities fit in the relationship between possibilities for professional development and organizational citizenship behavior.

Further analysis shows that demands-abilities fit plays a moderating role between various dimensions of job resources and OCB, specifically in social support (*β* = 0.077, *p* < 0.05), job control (*β* = 0.109, *p* < 0.01), and job development opportunities (*β* = 0.113, *p* < 0.01). When demands-abilities fit is high, it strengthens the positive promoting effect of social support, job control, and job development opportunities on OCB. The interaction terms “social support × demands-abilities fit,” “job control × demand-ability matching,” and “possibilities for professional development × demand-ability matching” explain 0.6% (Δ*R*^2^ = 0.006), 1% (Δ*R*^2^ = 0.01), and 1.2% (Δ*R*^2^ = 0.012) of the variance in OCB, respectively. As shown in Figures 3–5, compared to primary care staff with low demands-abilities fit, those with high demands-abilities fit exhibit a faster increase in OCB as social support, job control, and possibilities for professional development grow.

## 5 Discussion

### 5.1 Analysis of the status quo of primary care staffs’ OCB

The average score of the OCB of primary care staff in Guangzhou was 3.9, higher than the average 2.5, similar to the research results of scholars in other countries. Lucia Ratiu et al. (46) investigated the OCB of 545 primary care staff in two counties of a certain Eastern European country using the scale developed by Podsakoff et al. (57). The average score was 5.82, which was 3.5 higher than the average. Elavarasi et al. (47) investigated 400 medical workers from five hospitals in South India using the OCB scale compiled by Podsakoff et al. The average measurement score was 3.74, which is higher than the average score of 3.5. The OCB of medical workers in various countries is above the average level and remains at a relatively good level. The OCB of primary care staff in Guangzhou, China, is superior to that of medical workers in southern India. The results of this study show that people with authorized strength, older age, and higher professional titles exhibit better OCB, which may be because people without authorized strength face the threat of dismissal at any time and have a weak sense of organizational belonging. Owing to their rich work experience and longer working hours, people with junior professional titles devote more to their work than those of a younger age. Simultaneously, they will have a stronger sense of organizational belonging and thus exhibit better OCB.

### 5.2 Job resources positively influence OCB

The results of this study showed that job resources among primary care staff in Guangzhou positively influence their OCB. The higher the perceived level of job resources (*β* = 0.489, *p* < 0.001), the higher the OCB, explaining 23.7% of the variance in OCB. Further analysis of the dimensions of job resources follows.

### 5.3 Social support positively influences OCB

The results of this study show that the social support of primary care staff in Guangzhou positively influence OCB. The higher the perceived level of social support from colleagues and leaders (*β* = 0.175, *p* < 0.01), the higher the OCB. Social support has the greatest impact on the altruistic dimension of OCB (*β* = 0.327, *p* < 0.001). Social support refers to the material and emotional help obtained by individuals from social networks and involves the exchange of resources between people. Primary care staff’s perception of support or help from colleagues and leaders helps to reduce the pressure brought by high work requirements, generate more positive emotions, stimulate internal motivation, and return the other person’s help, thus showing more altruistic behavior. Wen et al. (48) proved that when organizational members receive support from the organization, leaders, and colleagues, their OCB also improves. Social support is also an important predictor of voice (*β* = 0.152, *p <* 0.01), consistent with the research conclusions of Griffin et al. (49), Van Dyne et al. (50), and other scholars on the voice of enterprise employees. Voice behavior is considered a risk-involving action and a high-resource-consuming behavior; however, the resource support brought by social support can offset part of the resource consumption associated with voice behavior (51). Consequently, organization members may be more daring and willing to speak up. Deng et al. (52) found that support from superiors and colleagues can reduce the workload of medical staff and significantly improve the service enthusiasm of medical staff.

### 5.4 Job control positively affects OCB

The results of this study showed that the higher the level of job control (*β* = 0.177, *p* < 0.01), the higher the OCB. Job control has the greatest influence on voice behavior in OCB (*β* = 0.252, *p* < 0.001). It refers to the autonomy of decision-making and the discretion of skills, reflecting how employees can influence their work behavior. SDT holds that people have intrinsic motivation and autonomy and pursue self-determination and self-actualization. Autonomy is a basic human need, which refers to individuals’ need to control their behavior and decision-making. When the need for autonomy is satisfied, the individual’s intrinsic motivation is stimulated (53). As knowledge workers, a strong sense of job control enables the primary care staff to satisfy their autonomy needs at work continuously, which helps to stimulate their intrinsic motivation and thus produce additional work behaviors, such as voice. Simultaneously, primary care staff with a high level of job control have a stronger sense of decision-making, and they take the initiative to think about how to do the work better to stimulate more voice behavior. Job control is also an important predictor of conscientiousness (*β* = 0.132, *p* < 0.05). Employees with a high level of job control have full autonomy in their work and realize that the quality of work results depends on their efforts. Therefore, employees will have a strong sense of accomplishment and responsibility for the success or failure of work. Examining medical staff in a German hospital, Boerner et al. (54) found that the sense of job control and workload are important factors affecting the OCB behavior of medical staff.

### 5.5 Possibilities for professional development positively influence OCB

The results of this study showed that possibilities for professional development (*β* = 0.203, *p* < 0.001) have a positive predictive effect on OCB, and possibilities for professional development had the greatest influence on conscientiousness in OCB (*β* = 0.281, *p* < 0.001). Possibilities for professional development refer to the organization’s provision of career development, training, promotion, and other employee opportunities. The possibilities for professional development of primary care staff are mainly reflected in the professional training opportunities or promotion incentive mechanisms provided by primary medical institutions. Owing to the growth expectation of personal growth, primary care staff expect to improve medical service through professional training, an important way to improve skills and a working resource. Possibilities for professional development, such as professional training and promotions, are often seen as a sign that the organization values them. When the personal growth needs of primary care staff are met, and they perceive that they are valued by the organization, based on the object consistency of social exchange relations, primary medical staff will return to the organization with higher work responsibility. Farid et al. (55) proved that when organizations provide possibilities for professional development, employees benefit from them and feel obligated to satisfy the company. Employees return what they get with extra-role behaviors, such as OCB.

Our research outcomes further confirm SET, demonstrating that primary care staff’s perception of organizational resources and support significantly influences their responses to the organization. When individuals obtain favorable possibilities for professional development, job control, and perceive strong social support from the organization, it fosters better OCB.

### 5.6 Demands-abilities fit positively regulates the relationship between job resources and OCB

This study shows that demands-abilities fit moderates job resources and OCB of primary care staff (*β* = 0.095, *p* < 0.01), with the interaction term explaining 0.08% of the variance in OCB (Δ*R*^2^ = 0.008). Moreover, it has a synergistic effect on the positive correlation between job resources and OCB. When the demands-abilities fit degree is high, the positive predictive effect of job resources on OCB is strengthened. Based on SDT, under a high degree of demands-abilities fit, the sense of competence of primary care staff is enhanced, their innate psychological needs of competence are satisfied, and they are willing to take the initiative to make use of job resources, fostering better OCB. Our study reveals the boundary conditions of demands-abilities fit on the impact of job resources on OCB, which constitutes a further contribution to the literature. Only a few studies have explored the moderating role of person-job fit in this process, while whether demands-abilities fit moderates the effect of job resources on OCB has not been sufficiently investigated. Additionally, it deepens the understanding of SDT.

### 5.7 Recommendations

Privacy protection during data collection and use is crucial. This study refers to the data classification standard, categorizes the data as moderately sensitive, employs anonymization techniques to safeguard personal privacy, adopts data quality control measures, and achieves a balance between privacy protection and data utility (56).

This study confirms that job resources can positively influence OCB. Therefore, primary healthcare institutions should provide more abundant job resources for primary care staff to promote OCB. First, they should emphasize support and encouragement from managers toward primary care staff. Since primary healthcare institutions often operate in team-based service models, attention should be paid to the collaborative relationship between general practitioners and nursing staff within the team, as well as inter-team cooperation, to build a stronger social support network and foster an organizational atmosphere of interpersonal support. Second, they should grant primary care staff greater job autonomy, adopting a results-oriented rather than process-oriented management approach, reducing unnecessary administrative interference. At the same time, they should establish a positive, open, and inclusive work environment to encourage primary care staff to participate in work-related decision-making, thereby enhancing their level of job control. Third, they should optimize the career advancement channels, establish a dedicated professional title evaluation system for primary levels, and provide more opportunities for promotion and career development for medical staff of different practice categories.

This study also confirms that demands-abilities fit plays a positive moderating role. Primary healthcare institutions prioritize allocating job resources to employees who possess a high demands-abilities fit. Meanwhile, through organizational training and incentive measures that encourage participation in such training, they enhance the abilities of primary care staff in the diagnosis and treatment of common diseases, the diagnosis, treatment, and management of chronic diseases, health management, health education, and the application of information technology. As the most fundamental units for infectious disease prevention and control, primary health service institutions need to enhance the diagnostic, treatment, preventive, and control capabilities, as well as the health emergency response capacities, of primary care staff in dealing with community infectious diseases. This aims to continuously improve the demands-abilities fit, adapt to the development needs of primary healthcare, and better leverage the positive role of job resources in OCB.

### 5.8 Limitations and future directions

This cross-sectional study was conducted in Guangzhou, Guangdong Province, China. The scope of the research investigation has certain limitations, and the generalizability of the survey results will be limited to some extent. The scope of the research objects can be expanded in the future. Furthermore, this study has a relatively small interaction coefficient between demands-abilities fit and job resources. Future studies should determine additional core and important moderating variables. Additionally, this study employed self-report surveys as the data collection method, which is currently a commonly used approach. Respondents may be influenced by social desirability bias and memory recall issues. Future research could explore diverse data collection methods, such as combining self-reports with supervisor evaluations or incorporating objective data to supplement self-reports, thereby reducing self-report bias and enhancing the credibility of research findings.

## 6 Conclusion

The results of this study show that the job resources of primary care staff have a positive predictive effect on OCB. Meanwhile, the demands-abilities fit plays a synergistic role in regulating the relationship between job resources and OCB. Therefore, primary health institutions can provide adequate job resources, create a conducive social support atmosphere between colleagues, leaders, and employees, and offer primary care staff appropriate job control and development opportunities. Furthermore, human resource management improves the level of staff demands-abilities fit, and enhances the OCB of primary care staff and improves their motivation, ultimately providing better primary health services for residents. This study provides valuable insights for developing countries on how to improve the work enthusiasm of primary care staff.

## Data Availability

The raw data supporting the conclusions of this article will be made available by the authors without undue reservation.
